# Patient-controlled analgesia morphine for the management of acute pain in the emergency department: a systematic review and meta-analysis

**DOI:** 10.1186/s12245-024-00615-3

**Published:** 2024-03-07

**Authors:** Muhammad Baihaqi Oon, Nik Hisamuddin Nik Ab. Rahman, Norhayati Mohd Noor, Mohd Boniami Yazid

**Affiliations:** 1 Department of Emergency Medicine, School of Medical Sciences, Universiti Sains Malaysia, Kubang Kerian, Kota Bharu, Kelantan, Malaysia; 2 Department of Family Medicine, School of Medical Sciences, Universiti Sains Malaysia, Kubang Kerian, Kota Bharu, Kelantan, Malaysia; 3 Hospital Universiti Sains Malaysia, Kubang Kerian, Kota Bharu, Kelantan, Malaysia

**Keywords:** PCA morphine, IV morphine, Acute pain, Emergency department

## Abstract

**Background:**

The ideal pain control approach is typically viewed as titration of analgesia for pain reduction and periodic pain evaluation. However, this method takes time and is not always possible in the crowded Emergency Department. Therefore, an alternative way to improve pain care in the Emergency Department is needed to avoid this unpleasant sensation in the patients. The best solution to tackle this situation is using Patient Controlled Analgesia (PCA), in the form of a PCA pump.

**Study objectives:**

This systematic review and meta-analysis was designated to evaluate the efficacy of PCA morphine in treating acute pain at Emergency Department.

**Methods:**

We searched databases Cochrane Central Register of Controlled Trials (CENTRAL), Medline, and Google Scholar up to February 2022 and identified randomized controlled trials with English language only that compare PCA morphine to IV morphine in treating patients presenting with acute pain at Emergency Department.

**Results:**

Eight trials were included in our review, comprising 1490 participants. We compared PCA morphine vs. IV morphine. There were no differences in the pain score between PCA and IV morphine (standard mean difference [SMD] = -0.20, *p* = 0.25). Further subgroup analyses (origin of the pain, time of assessment and the durations) showed no difference except for the dosages as the PCA morphine reduced the pain compared to IV morphine in low and high dosages but only two studies were involved. However, the analysis showed PCA morphine increased patient satisfaction and reduced the number of patients who required additional analgesia compared to IV morphine (MD 0.12, *P* < 0.001), (MD 0.47, *P* < 0.001) respectively. Data obtained in this review pertaining to adverse effects such as nausea, vomiting, pruritus, and drowsiness is limited since not all the trials reported the events.

**Conclusions:**

PCA morphine do appear to have a beneficial effect on the outcome of patient satisfaction and the number of patients who required additional analgesia. However, further studies targeting a larger sample size is required to increase the certainty of the evidence.

## Background

### Description of the condition

Pain is the most common reason that brings patients to the Emergency Department (ED). There is a study conducted in 2007 by Hospital Kuala Lumpur reported that approximately only 26.5% of 85% of the patients in moderate and severe pain received analgesia in the Emergency Department even though the pain scores were documented in the patient’s vital signs. This is an echo of what had been described from several studies conducted worldwide that revealed a large proportion of ED patients either receive no or sub-optimal analgesia [1–3]. We are all aware that when the patients do not receive adequate analgesia, it can lead to several negative impacts on the patient’s health, such as poor patient satisfaction and the possible risk of developing chronic pain [4].

The ideal pain control approach is typically viewed as titration of analgesia for pain reduction and periodic pain evaluation [5]. However, this method takes time and is not always possible in crowded emergency rooms. Therefore, an alternative way to improve pain care in the Emergency Department is needed to avoid this unpleasant sensation in the patients.

The best solution to tackle this situation is using Patient Controlled Analgesia (PCA), in the form of a PCA pump. PCA is considered the cornerstone of pharmacological management to improve and optimize pain in the emergency department. This device comprises volumetric pump that provides a predetermined intravenous dosage of medication when a control button is pressed. Anti-siphon and anti-reflux valves are also included in the PCA system to reduce the potential for unintentional medication distribution. The pump has additional features: a safety “lockout” feature that prevents it from delivering another dosage of opioids.

In post-operative treatment, many articles mentioned that PCA is widely employed. A Cochrane review of 49 randomized controlled trials (RCT) comparing PCA to conventional administration indicated that the PCA groups had better pain management and satisfaction [6]. However, in the acute care situation, there is a paucity of data regarding its efficacy and value which subsequently contribute to the fact that PCAs have not been widely implemented in the ED.

Several clinical trials and reviews have shown that when compared to the traditional approach of titrated bolus IV injection for the management of acute pain in the ED setting, PCA provides more effective pain relief and higher patient satisfaction [7–11].

### Description of the intervention

Patient Controlled Analgesia can be described as a technique based on a sophisticated microprocessor-controlled infusion pump that delivers a preprogrammed dose of opioids when the patient pushes a demand button. Historically, this method was first demonstrated by Roe in 1963. Subsequently, Philip H. Sechzer, considered a true pioneer of PCA, evaluated analgesic response to small IV doses of opioids given on patient demand by a nurse in 1968 and then by the machine in 1971. Over the years, the PCA method has gained popularity in many situations primarily related to the postoperative condition; however, with the tremendous evolvement of the technology sophistication, simplicity of use, versatility, and mobility, it has now become more feasible to be used in the Emergency Department for acute pain management.

There are many examples of opioids that are available in the market have been used successfully for PCA, such as morphine, fentanyl, meperidine, and others. However, morphine is the most researched and utilized [12, 13]. As the most studied and widely used intravenous PCA medication in many parts of the world, morphine remains the “gold standard.” It is worth noting that morphine contains an active metabolite, morphine-6-glucuronide (M6G), which causes analgesia, drowsiness, and respiratory depression. While morphine is removed mainly by glucuronidation, its active metabolite is mostly eliminated through renal excretion.

Besides, some of the most recent studies comparing PCA with a conventional method like intravenous injections have produced a contradictory result. Some showed significantly better analgesia and were more effective in managing the pain [7, 10, 11, 14]. Unlike the previous study, [15] revealed no difference between patient-controlled analgesia over usual ED care for acute pain management. Apart from that, the PCA also demonstrated that it leads to greater patient satisfaction when compared to the conventional method of titrated bolus intravenous injection [9–11, 16].

### How the intervention might work

The main advantage of using Patient Controlled Analgesia compared to the standard conventional titrated intravenous injection is that it offers better analgesic efficacy and experiences faster pain relief [8, 9, 16].On the other hand, more patients experienced greater satisfaction by using PCA than the usual method [7–9, 15, 16].

In addition, the number of patients who required additional analgesia after 120 min was significantly reduced in the patients who received PCA [14–16]. This showed how effective and superior PCA is compared to the conventional method.

### Why it is important to do this review

Although there is literature supporting the use of PCA post-operatively, there is a scarcity of evidence demonstrating its efficacy and value in the acute care context, which contributes to the fact that PCA has not been frequently used in the ED. Therefore, this review aims to analyze the benefit of PCA usage for acute pain relief in an Emergency Department setting. Several reviews have demonstrated the efficacy and safety of PCA. This review is critical when considering the numerous patients that come to the ED because of pain and the fact that most of them received suboptimal or inadequate pain management [1–3]. Therefore, if proven beneficial and feasible, this PCA method can become a new ‘game changer’ in managing acute pain in the Emergency Department.

## Methods

### Eligibility criteria

#### Types of studies

Randomized control trials (RCTs) comparing PCA morphine with the IV morphine for acute pain relief at Emergency Department. We included both blinded and open-label studies.

#### Types of participants

We included adult patients regardless of gender, ethnicity, co-morbidities or origin of pain.

#### Types of interventions

PCA morphine as an intervention. Comparison: IV morphine as standard care treatment.

#### Types of outcomes

Our outcome measures of interest were as mentioned below.

### Primary outcomes

Pain score.

Time to analgesia.

### Secondary outcomes

Patient satisfaction.

Adverse effects.

Length of stay at hospital.

Number of patients require additional analgesia.

### Search strategies

#### Electronic searches

We searched the Cochrane Central Register of Controlled Trials (CENTRAL 2022, Issue 1) and MEDLINE (1970- Feb 2022). We used the search strategy in Appendix 1 to search MEDLINE and CENTRAL. We adapted the search strategy for other databases. We restricted the publications to the English language only.

#### Searching other resources

We checked the reference list of identified RCTs and review articles to find unpublished trials or trials not identified by electronic searches. We searched for ongoing trials through the World Health Organization (WHO) International Clinical Trials Registry Platform (ICTRP) http://www.who.int/ictrp/en/ and www.clinicaltrials.gov.

### Trial selection

We scanned the titles and abstracts from the searches and obtained full-text articles when they appeared to meet the eligibility criteria or when there was insufficient information to assess the eligibility. We assessed the trials’ eligibility independently and documented the reasons for exclusion. We resolved any disagreements between the review authors by discussion. We contacted the authors if clarification is needed.

### Data extraction

Using data extraction form, from each of the selected trials we extracted study settings, participant characteristics (age, sex, ethnicity), methodology (number of participants randomized and analyzed, duration of follow-up), patient satisfaction, number of patients require additional analgesia, pain score, adverse effects, time to analgesia and length of stay at hospital.

### Risk of bias assessment

Risk of bias assessment for data quality performed using the Revised Cochrane risk of bias tool for randomized trial (RoB 2) version of 22 August 2019 [17]. We resolved any disagreements by discussion.

### Grading quality of evidence

We assessed the quality of evidence for primary and secondary outcomes according to GRADE methodology [18] for risk of bias, inconsistency, indirectness, imprecision, and publication bias; classified as very low, low, moderate, or high.

### Statistical analyses

#### Data synthesis

We planned to undertake meta-analyses using Review Manager 5.4 software (RevMan 2020) [32] and will use the random-effects model to pool data. Thresholds for interpreting of the I^2^ statistic can be misleading, since the importance of inconsistency depends on several factors. We planned to use the guide to the interpretation of heterogeneity as outlined: 0–40% might not be important; 30–60% may represent moderate heterogeneity; 50–90% may represent substantial heterogeneity; and 75–100% would be considerable heterogeneity [27].

#### Assessment of heterogeneity

We assessed the presence of heterogeneity in two steps. First, we assessed obvious heterogeneity at face value by comparing populations, settings, interventions, and outcomes. Second, we assessed statistical heterogeneity by means of the I^2^ statistic [27].

#### Measures of treatment effect

We measured the treatment effect for dichotomous outcomes using risk ratios (RRs) and absolute risk reduction, and for continuous outcomes we used mean differences (MDs); both with 95% confidence intervals (CIs). Continuous outcomes that were presented as the median with low and high end ranges/interquartile range were converted to the mean [28–31].

#### Subgroup analysis and investigation of heterogeneity

If possible, we conducted subgroup analyses on doses of morphine, origin of pain and time of assessment.

#### Unit of analysis issues

We checked included trials for unit of analysis errors. Unit of analysis errors can occur when trials randomized participants to intervention or control groups in clusters but analyzed the results using the total number of individual participants. We adjusted results from trials showing unit of analysis errors based on the mean cluster size and intracluster correlation coefficient [27].

#### Dealing with missing data

We contacted the original trial authors to request missing or inadequately reported data. We performed analyses on the available data in the event that missing data are not available.

#### Sensitivity analysis

We performed a sensitivity analysis to investigate the impact of risk of bias for sequence generation and allocation concealment of included studies.

#### Reporting biases

If there are sufficient studies, we used funnel plots to assess the possibility of reporting biases or small study biases, or both.

## Results

### Results of the search

**Fig. 1:**
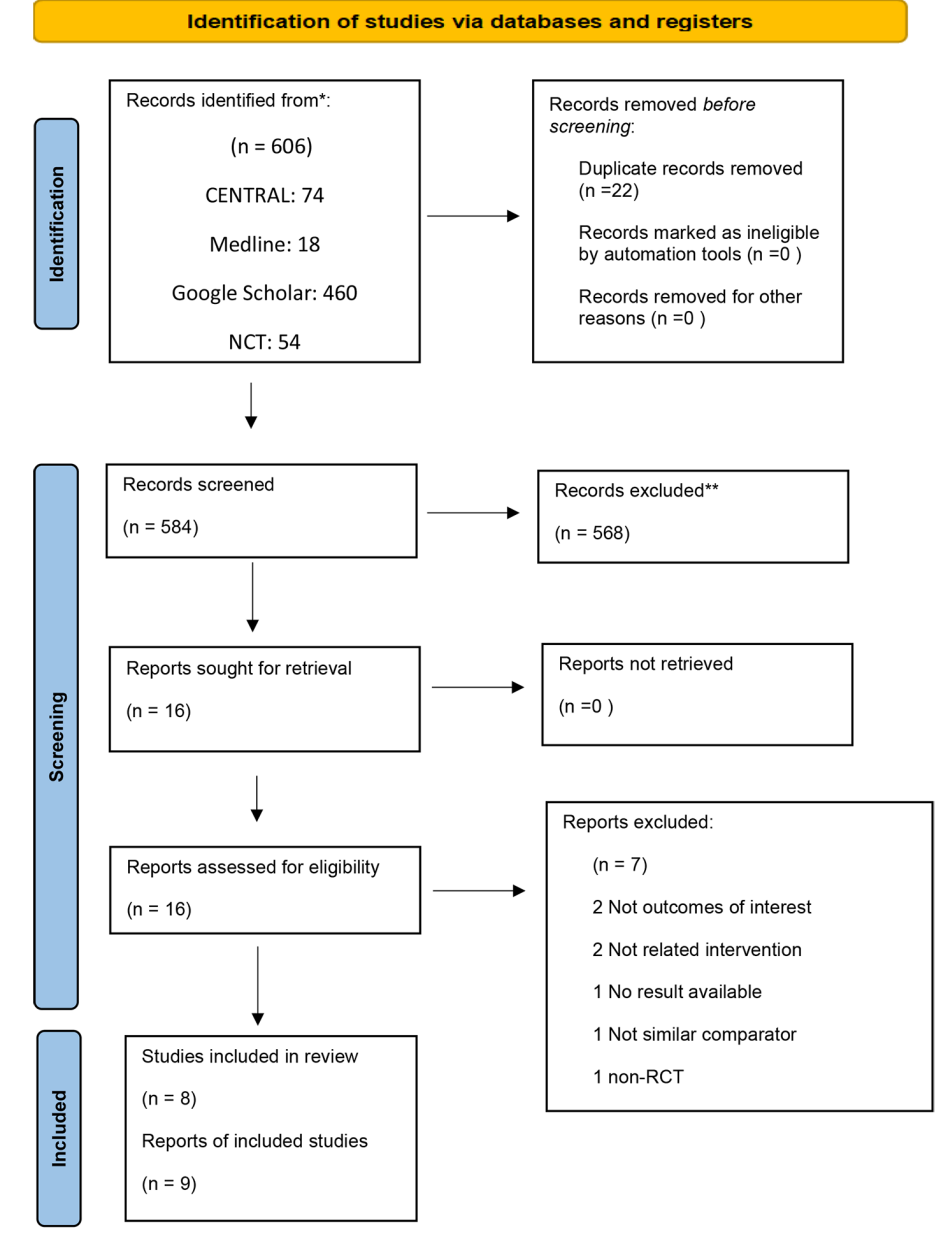
PRISMA study flow chart

We retrieved 606 records from the search of the electronic databases (Fig. 1). We screened a total of 584 records after duplicates were removed and 16 full copies were identified to possibly meeting the review’s inclusion criteria. Out of these 16 full copies, eight studies from nine reports were included in the review [7, 8, 10, 11, 14–16, 19]. We excluded seven articles from the review. Two articles were not outcome of interest [20, 21] and another two articles were not related intervention [22, 23]. One trial compared the efficacy of PCA morphine and PCA meperidine rather than PCA morphine vs. IV morphine [24]. One trial did not provide the result [33] and the other one was non-randomized trial [25]. In total, we included eight studies from nine reports in the review [7, 8, 10, 11, 14–16, 19]. Besides, there were two similar studies reported about patients presenting to the ED with traumatic pain requiring opioid analgesia, pain score of 7 or more on VAS and followed by subsequent [9, 19] reported about adult patients with acute traumatic pain (< 24 H) presenting to the ED with an initial pain score of 7 or more. In this case, Rahman and DeSilva [19] will be the reference citation.

### Included studies

We included eight trials with a total of 1490 participants [7, 8, 10, 11, 14–16, 19]. Three trials declared funding from a grant from the National Institute of Nursing Research (NINR) [15] and by the National Institute for Health Research (NIHR) [10, 11]. The other five trials declared no funding source [7, 8, 14, 16, 19].

### Participants

Seven of the eight trials were conducted in high-income countries [7, 8, 10, 11, 14–16]. One trial was conducted in low to middle-income countries [19]. All eight trials recruited participants from healthcare settings [7, 8, 10, 11, 14–16, 19]. Regarding the gender of participants, seven out of eight trials described this specific demographic factor in their trials [8, 10, 11, 14–16, 19]. In three trials, female participants were dominant [14–16]. In contrast, in the other four trials, male participants were dominant [8, 10, 11, 19]. The gender of participants was not mentioned in one trial [7]. Three trials included participants with pain from non-traumatic origin [7, 10, 14] while three other trials included participants with pain from a traumatic origin [8, 11, 19]. In addition, two trials did not mention or specify the origin of pain [15, 16].

### Interventions

All eight trials administered PCA morphine 1 mg [7, 8, 10, 11, 14–16, 19]. Still, the two trials [7, 16] further specified PCA morphine into high doses of 1.5 mg and 2.7 mg, respectively.

In comparison to the intervention group, the control group in seven trials received intravenous morphine [7, 8, 10, 11, 15, 16, 19]. However, one trial received a continuous infusion of morphine [14].

### Outcomes

All eight trials reported that they had measured pain as the primary outcome [7, 8, 10, 11, 14–16, 19]. Three main instruments were used in these trials to quantify the pain which were Verbal Pain Score (VPS) [7], Visual Analog Scale (VAS) [8, 10, 11, 14, 19] and Numeric Rating Scale (NRS) [15, 16]. Three trials included participants with pain from a non-traumatic origin [7, 10, 14], while three other trials included participants with pain from a traumatic origin [8, 11, 19]. In addition to that, two trials did not mention or specified regarding the origin of pain [15, 16], two trials did not mention or specify the origin of pain [15, 16]. Two trials mentioned about low and high dose [7, 16].

Six trials measured the pain at 120-minute intervals [8, 10, 11, 15, 16, 19], but two trials measured the pain at 8 and 48 h respectively [7, 14]. The other primary outcome was time to analgesia, which was the time from arrival to the randomization measured in minutes reported in three trials [10, 11, 15].

Our secondary outcomes were patient satisfaction, which was obtained from a questionnaire after completing the treatment [7, 8, 10, 11, 15, 16, 19]. Likert scale was used to obtain patient satisfaction in three trials [10, 11, 19], while one trial used dichotomous scale [7]. However, two trials did not describe specifically the tool to get the questionnaire [8, 15]. The next secondary outcomes were length of hospital stay which is described as the number of days in which patients stayed in the hospital [7, 10, 11, 14], number of patient require additional analgesia after 120 min [14–16] and adverse effects which comprise of nausea/vomiting [7, 8, 10], nausea [14–16, 19], vomiting [15, 16, 19], pruritus [7, 10, 14–16] and drowsiness [7, 8, 10, 14–16, 19] (Table 1).

**Table 1:** Characteristics of included studies

Reference	No of patients	Population	Age	Outcomes
[7]	45	Patient presented to ED with sickle cell crisis pain	18–65	• Pain score • Patient satisfaction • Adverse effects • Length of stay
[8]	86	Patient presented with acute traumatic pain presenting to the ED with an initial pain score of 7 or more	> 16	• Pain score • Patient satisfaction • Adverse effects
[14]	25	Patient presented with vaso-occlusive crisis in sickle cell disease	> 17	• Pain score • Adverse effects • Length of stay • Number of patients require additional analgesia
[16]	206	Patient presented with acute abdominal pain in the ED	18–65	• Pain score • Patient satisfaction • Adverse effects • Number of patients require additional analgesia-
[19]	96	Patient presented to the ED with acute traumatic pain (< 24 h) with an initial pain score of 7 or more	18–55	• Pain score • Patient satisfaction • Adverse effects
[11]	200	Patients presented to the ED with traumatic musculoskeletal injury requiring IV analgesia	18–75	• Pain score • Time to analgesia • Patient satisfaction • Adverse effects • Length of stay
[10]	196	Patients presented to the ED with non-traumatic abdominal pain requiring IV analgesia	18–75	• Pain score • Time to analgesia • Patient satisfaction • Adverse effects • Length of stay
[15]	636	Patients presented with acute pain to the ED	18–65	• Pain score • Adverse effects • Length of stay • Number of patients require additional analgesia

### Risk of bias of included studies

**Fig. 2:**
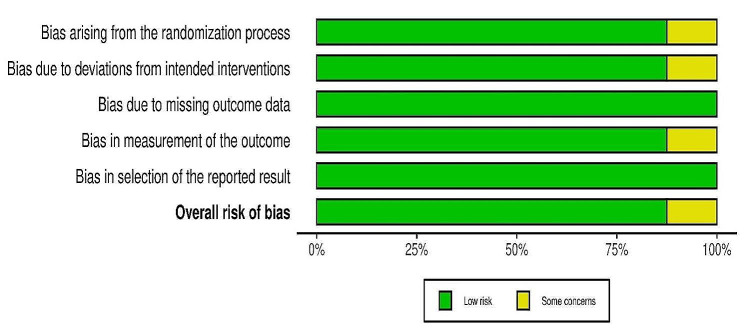
‘Risk of bias’ graph: review authors’ judgements about each risk of bias item presented as percentages across all included studies

**Fig. 3:**
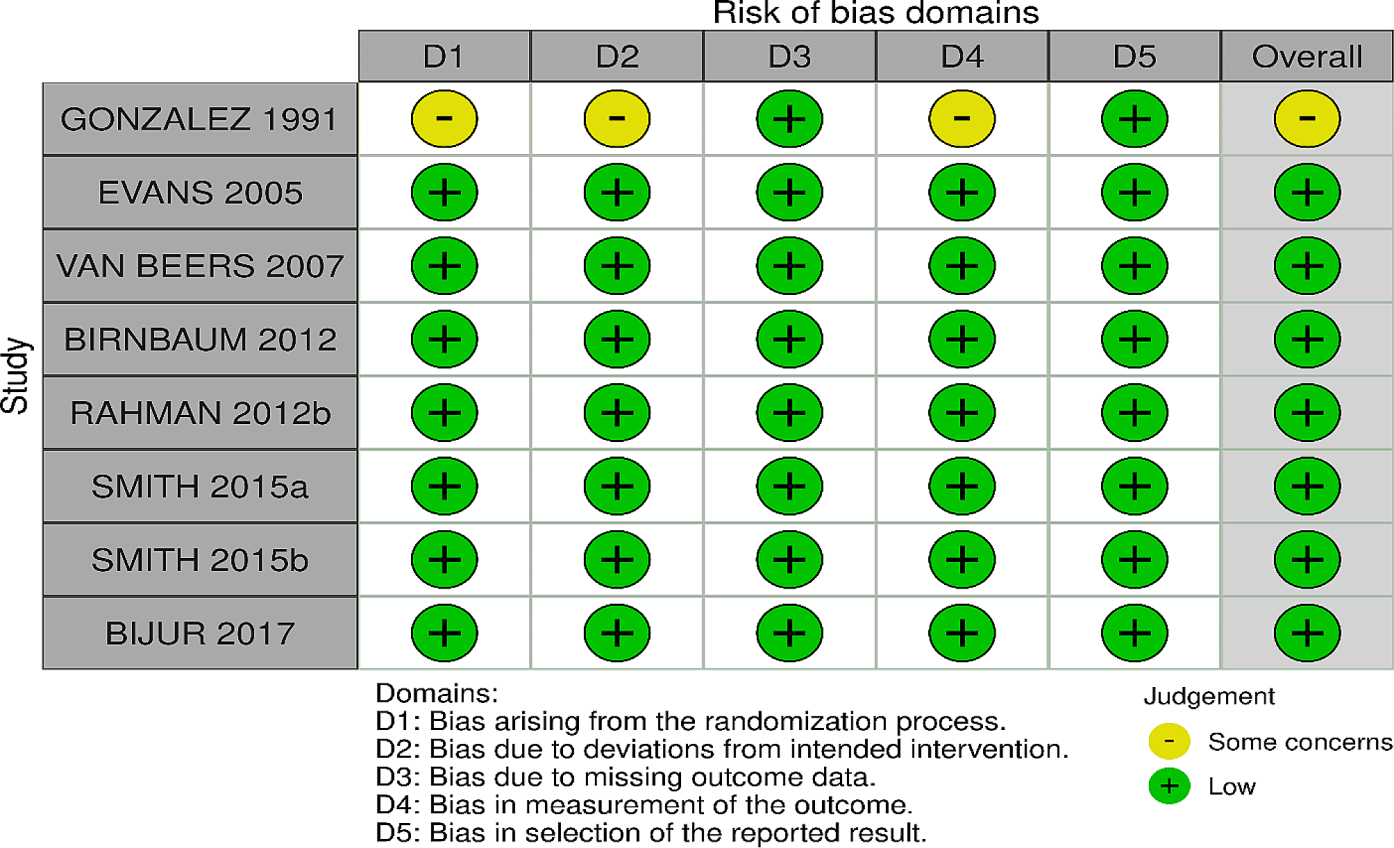
‘Risk of bias’ summary: review authors’ judgments about each risk of bias item for each included study

The assessment of risk of bias is shown in Figs. 2 and 3. Figure 2 shows the proportion of studies assessed as low, high or unclear risk of bias for each risk of bias indicator. Figure 3 shows the risk of bias indicators for individual studies.

### Randomization and allocation concealment

Seven trials described the method of randomization used and were judged as low risk. Four trials randomized the participants according to a computer-generated sequence [8, 10, 11, 19], three trial using blocks [14–16] and one trial did not describe the method of randomization [7]. Allocation concealment was described in seven trials. All seven trials used a sealed, opaque envelope technique [8, 10, 11, 14–16, 19]. The method for allocation concealment was not reported in one trial [7]. Thus, we judged the allocation concealment as some concerns.

### Blinding

The investigators and participants were not blinded in all eight trials [7, 8, 10, 11, 14–16, 19]. Blinding was not considered desirable or feasible in the study for several reasons. First, subjects’ experience with the modality of analgesia administration itself (patient-controlled analgesia versus usual care) could be accomplished only if patients were aware of the treatment assignment. Second, blinding would require subjects in significant pain to receive sham dosing on a patient-controlled analgesia pump, which was thought unethical. Physician blinding would require decisions about additional analgesic administration to be made without knowing whether the active drug was simultaneously available for self-administration. Third, blinding was not possible for this study owing to the nature of the intervention. However, because most of the outcomes were objectively assessed, we judged the risk of bias as low because the outcome measurements were unlikely to be influenced.

### Incomplete outcome data

All eight trials [7, 8, 10, 11, 14–16, 19] carried out an intention-to-treat analysis in which the participants were analyzed according to the groups they were initially assigned. The incomplete outcome data was judged as low risk in three trials. In five trials [7, 10, 11, 15, 19] all participants completed the trial and were included in the analysis. In three trials [8, 14, 16], some participants were not analyzed due to missing data [8]. reported that seven participants did not complete the trial [14], reported two participants withdrew their consent while Birnbaum, Schechter [16] reported that 5 participants had to be excluded from the analysis for specific reasons mentioned. However, we concluded the incomplete outcome data bias to be low for these trials because the proportion of missing data was small and evenly distributed between the intervention and control groups.

### Selective reporting

All eight trials reported the outcomes as specified in their methods section and were judged as low risk [7, 8, 10, 11, 14–16, 19].

### Other potential source of bias

We detected no other potential source of bias in all eight trials and were judged as low risk [7, 8, 10, 11, 14–16, 19].

### Effects of interventions

**Primary outcomes**.

1. Pain

**Fig. 4:**
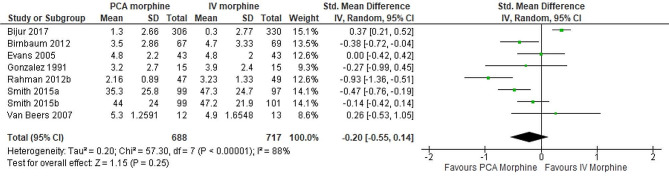
PCA morphine vs. IV morphine for the outcome of pain

Analysis of eight trials Gonzalez et al., 1991, Evans et al., 2005, van Beers et al., 2007, Rahman and DeSilva, 2012b, Birnbaum et al., 2012, Smith et al., 2015b, Smith et al., 2015a, Bijur et al., 2017) showed there was no difference between PCA morphine and IV morphine (SMD − 0.20; 95% CI -0.55 to 0.14; I^2^ statistics = 88%; P 0.25; eight trials;1405 participants; moderate quality evidence) (Fig. 4; Table 2).

**Table 2:** GRADE assessment for PCA morphine compared to IV morphine

Outcomes	№ of participants (studies) Follow-up	Certainty of the evidence (GRADE)	Relative effect (95% CI)	Anticipated absolute effects	
				Risk with IV morphine	Risk difference with PCA morphine
Patient satisfaction	1166 (5 RCTs)	⨁⨁⨁⨁ High	not estimable	734 per 1,000	**734 fewer per 1,000** (734 fewer to 734 fewer)
Number of patients requiring additional analgesia	760 (3 RCTs)	⨁⨁⨁◯ Moderate^a^	**OR 0.47** (0.34 to 0.64)	387 per 1,000	**158 fewer per 1,000** (210 fewer to 99 fewer)
Adverse effect: Nausea/Vomiting	684 (5 RCTs)	⨁⨁⨁◯ Moderate^a^	**OR 1.25** (0.82 to 1.90)	214 per 1,000	**40 more per 1,000** (32 fewer to 127 more)
Pain	1405 (8 RCTs)	⨁⨁⨁◯ Moderate^b^	-	-	SMD **0.2 lower** (0.55 lower to 0.14 higher)
Pain by dosage - Low dosage (less than 1 mg)	166 (2 RCTs)	⨁⨁⨁◯ Moderate^a^	-	-	SMD **0.36 lower** (0.67 lower to 0.06 lower)
Pain by dosage - High dosage (more than 1 mg)	179 (2 RCTs)	⨁⨁⨁◯ Moderate^a^	-	-	SMD **0.38 lower** (0.68 lower to 0.09 lower)
Pain by intervals - Pain measured at 120 min	868 (3 RCTs)	⨁⨁⨁◯ Moderate^b^	-	-	SMD **0.3 lower** (1.08 lower to 0.49 higher)

*RCT* randomized controlled trial, *PCA* patient-controlled analgesia, *IV* intravenous, *CI* confidence interval, *MD* mean difference, *OR* odds ratio, *SMD* standardised mean difference, *a* The number of events less than 400, *b* I square more than 50%

**Fig. 5:**
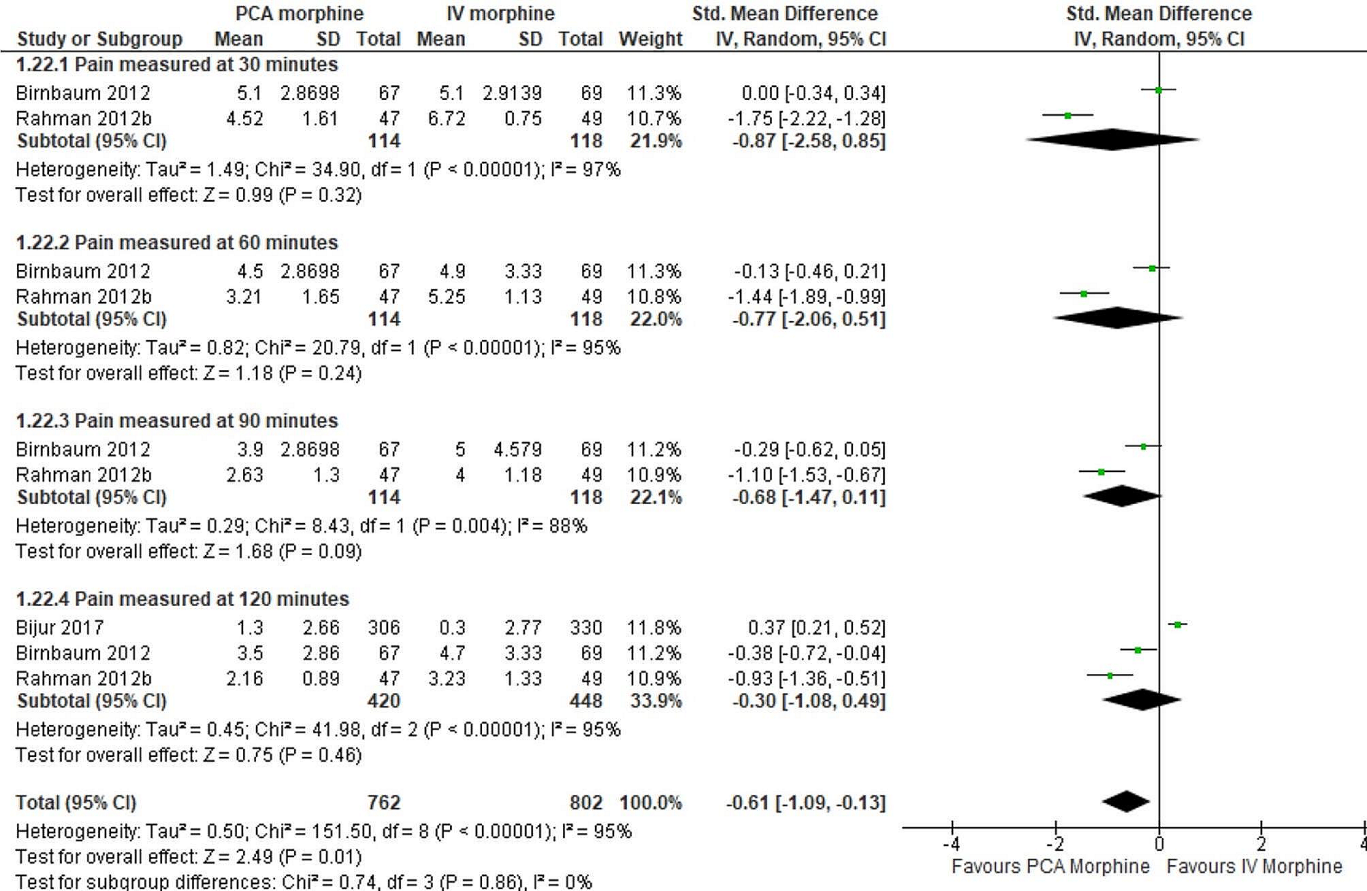
PCA morphine vs. IV morphine for the outcome of pain by 30 min intervals

A subgroup analysis by time of assessment was performed. There was no difference in pain between PCA morphine and IV morphine assessed at 30 (SMD − 0.87; 95% CI − 2.58 to 0.85; I^2^ statistics = 97%; P 0.32; two trials; 232 participants; low quality evidence), 60 (SMD − 0.77; 95% CI -2.06 to 0.51; I^2^ statistics = 95%; P 0.24; two trials; 232 participants; low quality evidence), 90 (SMD − 0.68; 95% CI -1.47 to 0.11; I^2^ statistics = 88%; P 0.09; two trials,232 participants; low quality evidence) and 120 (SMD − 0.30; 95% CI -1.08 to 0.49; I^2^ statistics = 95%; P 0.46; three trials,868 participants; moderate quality evidence) minutes (Fig. 5; Table 2).

**Fig. 6:**
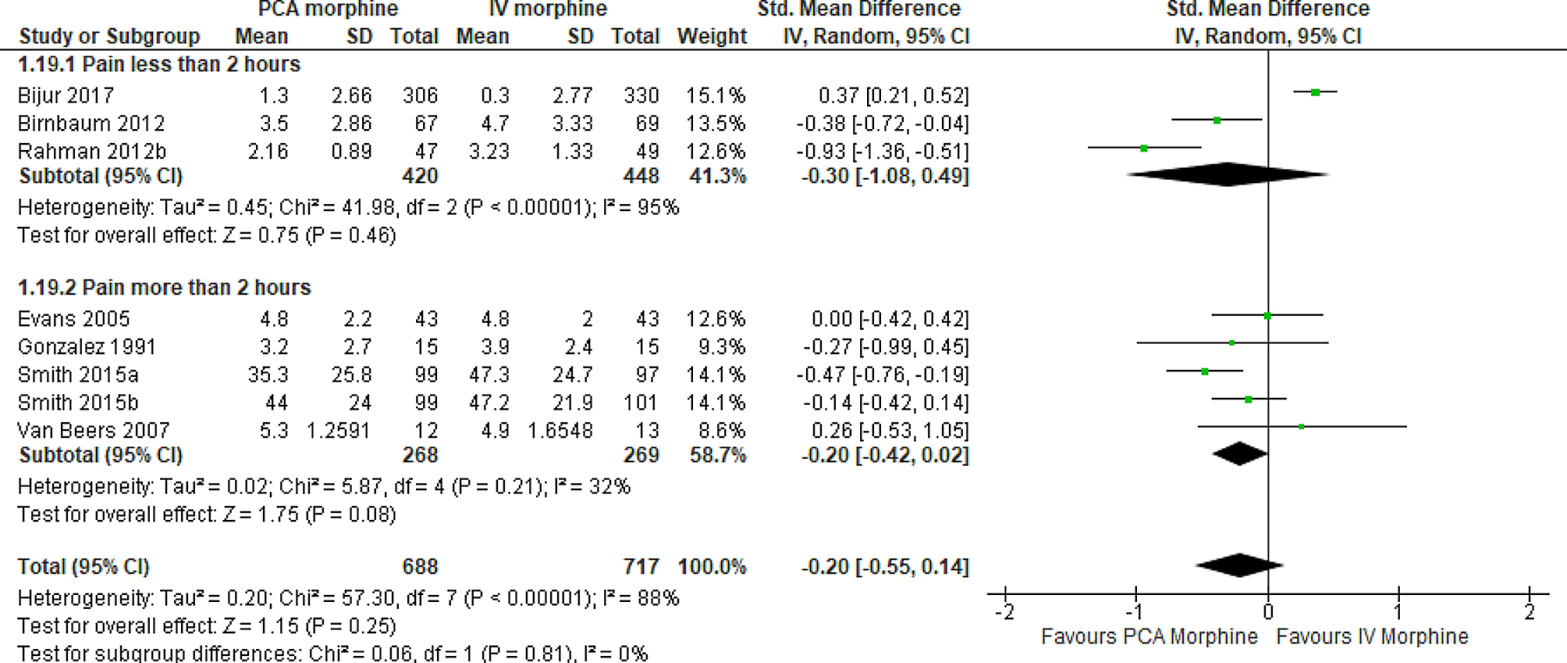
PCA morphine vs. IV morphine for the outcome of pain by duration

Then subgroup analysis by the durations was performed. At pain less than 2 hours (SMD − 0.30; 95% CI -1.08 to 0.49; I^2^ statistics = 95%; P 0.46; three trials,868 participants; moderate quality evidence) and more than 2 hours (SMD − 0.20; 95% CI -0.42 to 0.02; I^2^ statistics = 32%; P 0.08; five trials,537 participants; moderate quality evidence), there was no difference between the two groups (Fig. 6; Table 2).

**Fig. 7:**
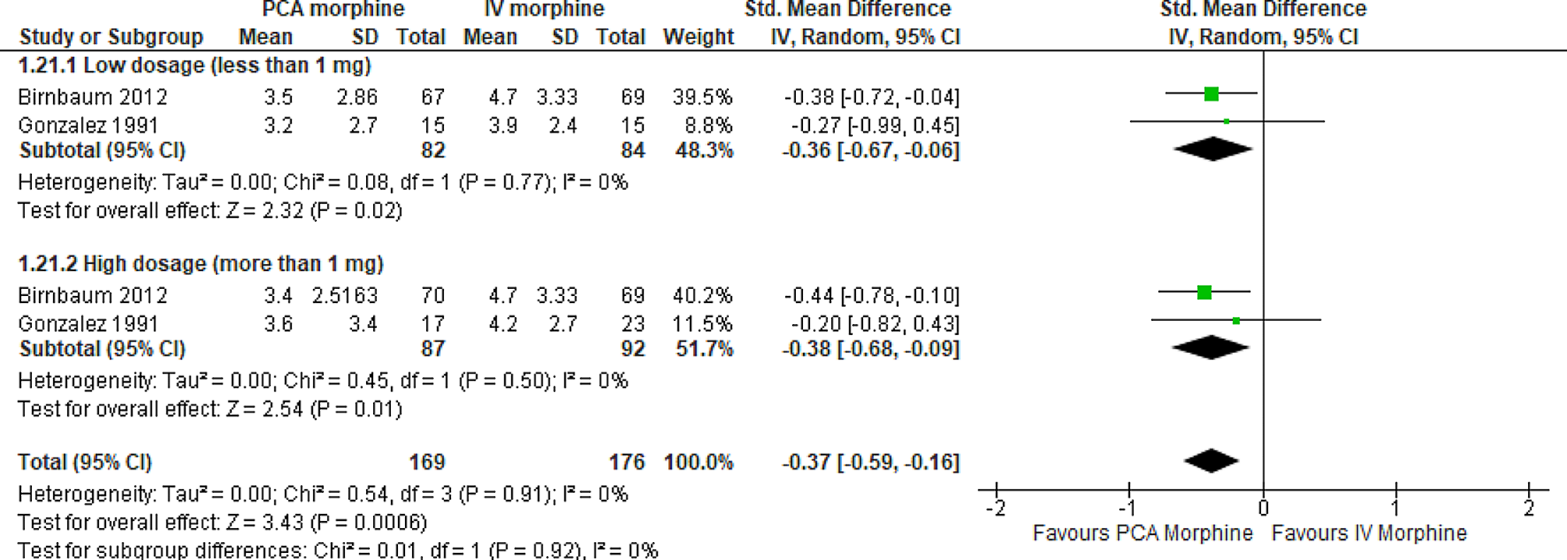
PCA morphine vs. IV morphine for the outcome of pain by dosages

The subgroup analysis by dosages was performed. However, only two studies [7, 16] reported the dosage involved; therefore, they could still not explain the high heterogeneity between studies for the pain outcome. At a dosage less than 1 mg (SMD − 0.36; 95% CI -0.67 to -0.06; I^2^ statistics = 0%; P 0.02; two trials,166 participants; moderate quality evidence) and more than 1 mg (SMD − 0.38; 95% CI -0.68 to -0.09; I^2^ statistics = 0%; P 0.01; two trials,179 participants; moderate quality evidence), PCA morphine reduced pain more than IV morphine (Fig. 7; Table 2).

**Fig. 8:**
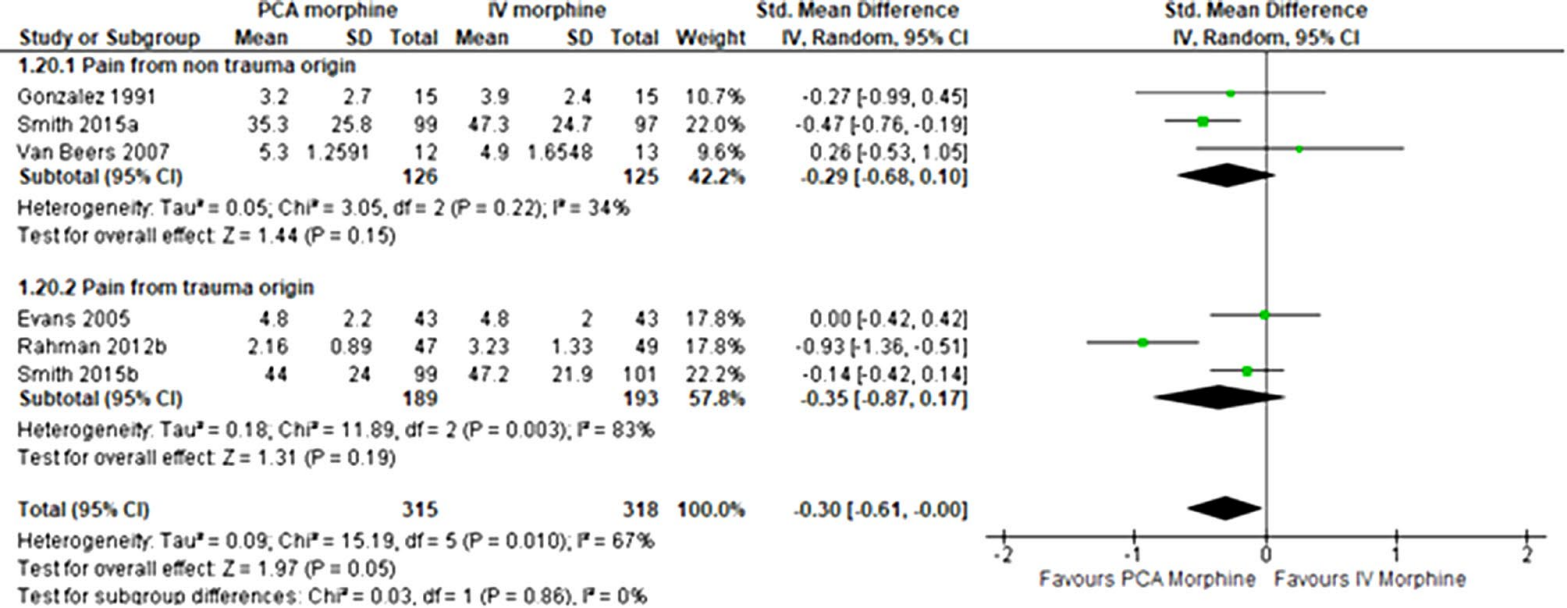
PCA morphine vs. IV morphine for the outcome of pain by origin

The subgroup analysis by the origin of pain was performed. The pain from non-trauma origin showed no difference between the two groups (SMD − 0.29; 95% CI -0.68 to 0.10; I^2^ statistics = 34%; P 0.15; three trials,251 participants; moderate quality evidence) (Fig. 8). Besides, the pain from trauma origin also showed no difference between the two groups (SMD − 0.35; 95% CI -0.87 to 0.17; I^2^ statistics = 83%; P 0.19; three trials,382 participants; low quality evidence) (Fig. 8).

2. Time to analgesia

**Fig. 9:**
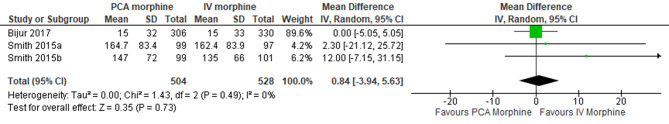
PCA morphine vs. IV morphine for the outcome of time to analgesia

Analysis of three trials [10, 11, 15] showed there was no difference between the two groups (MD 0.84; 95% CI -3.94 to 5.63; I^2^ statistics = 0%; P 0.73; three trials, 1032 participants; high quality evidence) (Fig. 9).

### Secondary outcomes

1. Patient satisfaction

**Fig. 10:**
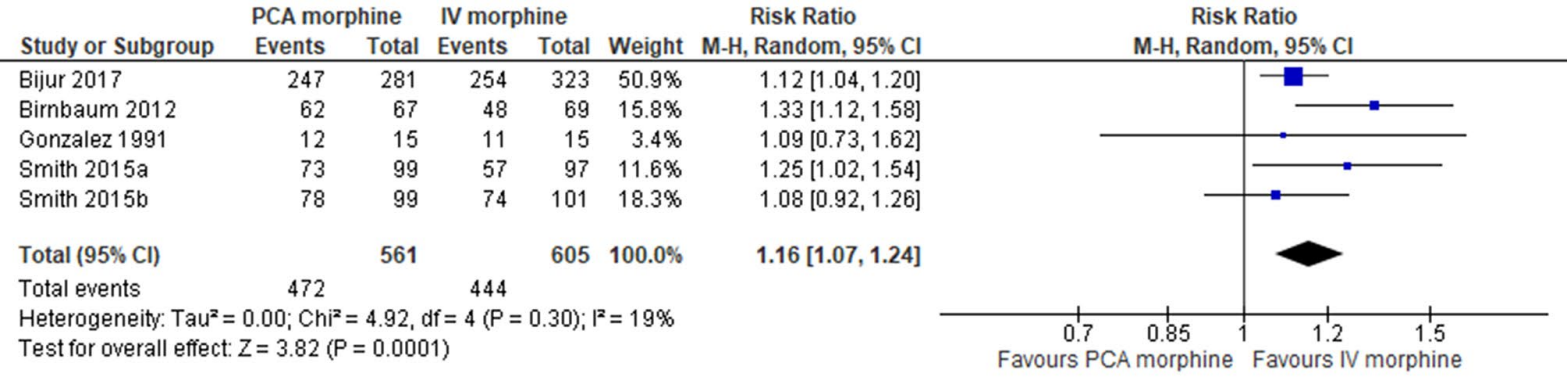
PCA morphine vs. IV morphine for the outcome patient satisfaction

Five trials reported on patient satisfaction [7, 10, 11, 15, 16]. The analysis showed PCA morphine increased patient satisfaction compared to IV morphine (MD 0.12; 95% CI 0.06 to 0.17; I^2^ statistics = 21%; *P* < 0.001; five trials; 1166 participants; high quality evidence) (Fig. 10; Table 2).

2. Number of patients requiring additional analgesia

**Fig. 11:**
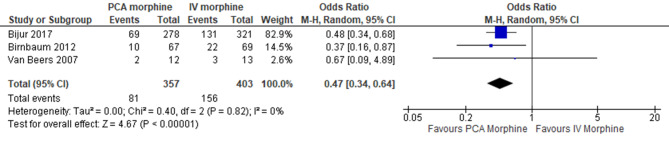
PCA morphine vs. IV morphine for the outcome number of patients requiring additional analgesia

Three trials reported on the patients requiring additional analgesia [14–16]. The result showed PCA morphine reduced the number of patients who required additional analgesia compared to IV morphine (MD 0.47; 95% CI 0.34 to 0.64; I^2^ statistics = 0%; *P* < 0.001; three trials; 760 participants; moderate quality evidence) (Fig. 11; Table 2).

3. Length of stay

**Fig. 12:**
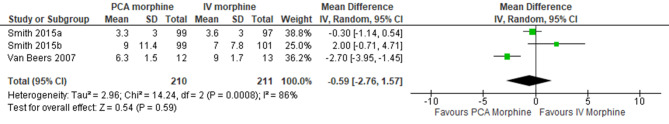
PCA morphine vs. IV morphine for the outcome length of stay

Data from three studies were pooled together to assess the length of stay [10, 11, 14]. The result showed no difference between the groups (MD-0.59; 95% CI -2.76 to 1.57; I^2^ statistics = 86%; P 0.59; three trials; 421 participants; low quality evidence) (Fig. 12).

4. Adverse effect: Nausea/Vomiting

**Fig. 13:**
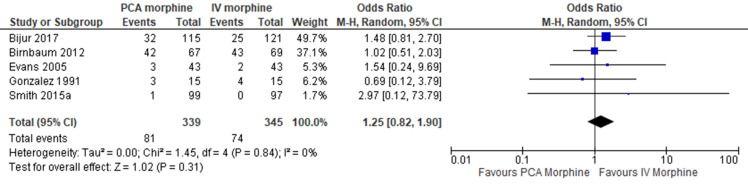
PCA morphine vs. IV morphine for the outcome adverse effect of nausea/vomiting

Five trials reported the adverse effect of nausea/vomiting [7, 8, 10, 15, 16]. The result showed no difference between the two groups (MD 1.25; 95% CI 0.82 to 1.90; I^2^ statistics = 0%; P 0.31; five trials; 684 participants; moderate quality evidence) (Fig. 13; Table 2).

5. Adverse effect: Pruritus

**Fig. 14:**
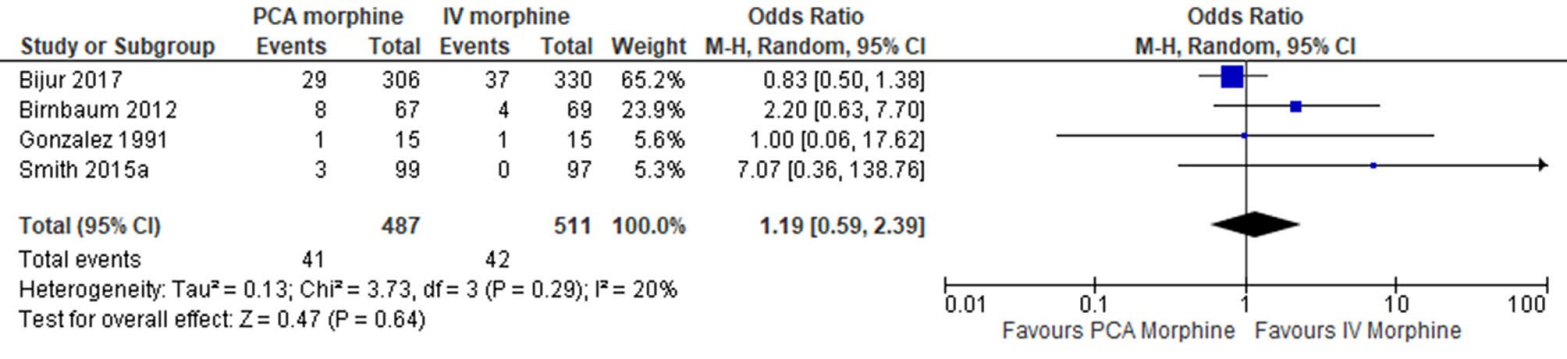
PCA morphine vs. IV morphine for the outcome adverse effect of pruritus

Four trials reported the adverse effect of pruritus [7, 10, 15, 16]. The result showed no difference between the two groups (MD 1.19; 95% CI 0.59 to 2.39; I^2^ statistics = 20%; P 0.64; four trials; 998 participants; moderate quality evidence) (Fig. 14).

6. Adverse effect: Mild sedation/Drowsiness

**Fig. 15:**
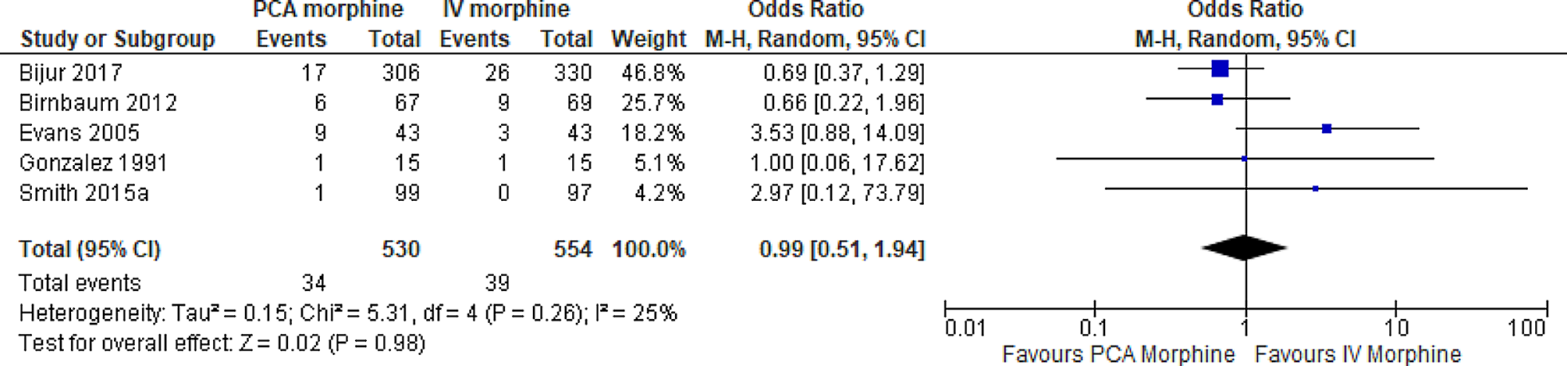
PCA morphine vs. IV morphine for the outcome adverse effect of mild sedation/drowsiness

Five trials reported the adverse effect of mild sedation/drowsiness [7, 8, 10, 15, 16]. The result showed no difference between the two groups (MD 0.99; 95% CI 0.51 to 1.94; I^2^ statistics = 25%; P 0.98; five trials; 1084 participants; moderate quality evidence) (Fig. 15).

7. Adverse effect: Constipation

There was only one trial that reported on this outcome [14] (one trial; 25 participants). The study only gave value area under the curve, which made it unable to analyze.

## Discussion

### Summary of main results

This review was designed to include all randomized controlled trials (RCTs) addressing the effectiveness of PCA morphine for the management of acute pain in the Emergency Department. There was no difference in the pain score between the PCA morphine group and IV morphine group. The subgroup analyses (i.e. origin of the pain, time of assessment and the durations) showed no difference except for the dosages as the PCA morphine reduced the pain compared to IV morphine in low and high dosages. However, only two studies were involved in this subgroup analysis of dosages and were still unable to explain the high heterogeneity between studies for the pain outcome.

More patients showed satisfaction with PCA morphine compared to IV morphine. Besides, PCA morphine also reduced the number of patients requiring additional analgesia. Adverse events including nausea and vomiting, pruritus and drowsiness were reported by a combination of five trials. However, for most trials, the predetermined adverse events did not show any difference between the two groups.

### Overall completeness and applicability of evidence

We performed a comprehensive and systematic literature review to assess the effectiveness of PCA morphine for management of acute pain. We searched trial registers to find potentially relevant trials. Although we attempted to be as inclusive as possible in our searches, the literature we identified was predominantly published in English. We included eight trials, but the findings of this review may not be applicable to children as they were excluded from the included trials. All the trials included in the review were conducted at Emergency Department. We could not be sure whether the findings of this review were applicable in other environments such as in the setting of intensive care units or post operative procedures.

For the primary outcome of pain, there are substantial differences among the included trials with regards to the tools to quantify the pain. Three main instruments was used in these trials to quantify the pain which were VPS [7],VAS [8, 10, 11, 14, 19] and NRS [15, 16]. This may introduce heterogeneity and may limit the applicability of the findings. However, we were able to conduct several subgroup analyses for this primary outcome. From the reported incidence of adverse events, we were able to detect common side effects, that is nausea, vomiting, pruritus, and drowsiness. The information on adverse events came from 5 trials involving about 684 participants, but there was a lack of information on more rare and serious adverse events. We could not include constipation in the adverse effects, hence do the analysis due to only one trial reported for this adverse effect.

### Quality of the evidence

Using the GRADE approach, we assessed the overall level of evidence contributing to this review as low to high quality. In the meta-analysis of predetermined outcomes, we encountered a certain degree of heterogeneity. Some outcomes showed substantial heterogeneity. We downgraded our primary outcome due to concerns about this since all trials contributing to the outcome of pain showed the high heterogeneity. Similarly, we also encountered significant heterogeneity due to I2 value more than 50% or imprecision as a results of small sample size in the meta-analysis of subgroup analysis of primary outcomes. As such, we decided to downgrade the certainty. Besides that, we judged and downgraded the evidence for frequency of adverse effects to be a low quality due to imprecision as a results of small sample size.

Generally, we assessed the risk of bias for most trials as either low or some concern for most domains. The risk of bias for method of randomization and allocation concealment were some concerns for one trial [7] as there was no mention on the technique used from the trial manuscripts. However, given that the nature of the outcomes measured were objective rather than subjective, they were unlikely to be influenced. Three trials [8, 14, 16] were also judged to have some concern of bias concerning incomplete outcome data but we concluded the incomplete outcome data bias to be low for these trials because the proportion of missing data was small and evenly distributed between the intervention and control groups.

### Potential biases in the review process

We were aware of the possibility of introducing bias at every stage of the review process. Following the Cochrane methods, we developed a comprehensive search strategy across multiple databases to capture all eligible trials. We strived to reduce publication bias by searching different databases and examining the reference lists of all linked articles for additional references. We cannot, however, guarantee that we have discovered all the trials in this field. Despite the extensive search strategy, we only included trials published in English language. As a result, certain studies done in other languages may have been overlooked. Two review authors were appointed to assess the study eligibility, conduct data extraction, and evaluate the risk of bias in the included trials independently to reduce the potential bias in the review process. Any disagreements would be resolved with discussion. Although there were eight trials included in this review, not all trials reported all outcomes and some of the trials reported the outcomes in a non-usable format, hence the data could not be analysed.

### Agreements and disagreements with other studies or reviews

One systematic review has examined the effectiveness of PCA for management of acute pain in Emergency Department [26]. This review comprised of ten studies [7–11, 15, 16, 19, 24, 25] but we only included seven of these in our review. This systematic review has demonstrated that ED use of PCA therapy is associated with increased patient satisfaction, decreased pain scores, and an overall increase in opioid consumption. We consider our review to be consistent with the findings of other reviews that assessed the effectiveness of PCA for management of acute pain in Emergency Department. However, our findings may be limited due to the substantial heterogeneity encountered in the meta-analysis and the small population size.

## Conclusion

### Implications for practice

We found low to moderate quality evidence to support the effectiveness of PCA morphine for management of acute pain relief. Although there were inconsistencies in the findings amongst these studies, as well as important differences in study methods and outcome measures used, PCA morphine do appear to have a beneficial effect on the outcome of patient satisfaction and the number of patients who required additional analgesia. However, variable factors in this review, including population size, settings, and durations used, would need to be considered when interpreting the findings. Data obtained in this review pertaining to adverse effects such as nausea, vomiting, pruritus, and drowsiness is limited since not all the trials reported the events. Overall, we found evidence in this review that these studies do show some effectiveness and benefit that suggest PCA is a reasonable therapy choice for select patient populations provided the important stakeholders such as the patients, nurses, and doctors are comfortable with their use to support its widespread use in the Emergency Department.

### Implications for research

Further studies focusing on the limitations faced in our review are warranted to address the research question. More research targeting a larger sample size is required to enable a precise analysis and to increase the certainty of the evidence of the outcomes. Well-designed studies conducted in the emergency department are still needed to evaluate the ideal patient population to whom these PCA may provide the most benefit as well as a systematic and robust cost-analysis to ensure the feasibility of use in the future. We also encouraged future trials to explore further the effectiveness of PCA morphine in the other special population, such as the paediatric age group and pregnant women, as pain may also occur among them. If future studies are to be conducted, a standard time of assessment and similar tools measurement on pain intensity and improvement should be used. The data on each adverse effect that may occur with medications used, need for additional analgesia, recurrence of pain, length of ED stay, and comorbidities should also be collected and explored further. These findings are very crucial that will help to provide insight into how PCA morphine can be used to aid in managing acute pain in Emergency Department.

## Data Availability

All data generated or analysed during this study are included in this published article.

## Appendix 1: search strategy

COCHRANE/CENTRAL

1) patient control analgesia morphine.

2) acute pain.

3) adult patient.

4) emergency department.

5) #1 AND #2 AND #3 AND #4.

MEDLINE/PUBMED

pca morphine AND emergency department.

GOOGLE SCHOLAR

Words: analgesia.

Phrase: patient controlled.

Without words:surgery OR block.

1990–2021.

NCT

Condition: acute pain.

Other term: pca morphine.

*interventional.

*population:adult and older.

*Completed and recruiting.
