# Correction: Patient-controlled analgesia morphine for the management of acute pain in the emergency department: a systematic review and meta-analysis

**DOI:** 10.1186/s12245-024-00625-1

**Published:** 2024-04-10

**Authors:** Muhammad Baihaqi Oon, Nik Nik Ab. Rahman Hisamuddin, Norhayati Mohd Noor, Mohd Boniami Yazid

**Affiliations:** 1 Department of Emergency Medicine, School of Medical Sciences, Universiti Sains Malaysia, Kubang Kerian, Kota Bharu, Kelantan Malaysia; 2 Department of Family Medicine, School of Medical Sciences, Universiti Sains Malaysia, Kubang Kerian, Kota Bharu, Kelantan Malaysia; 3 Hospital Universiti Sains Malaysia, Kubang Kerian, Kota Bharu, Kelantan Malaysia

**Correction: Int J Emerg Med 17, 37 (2024)**

10.1186/s12245-024-00615-3

In this article [1], the wrong figure appeared as Fig. 8.

The incorrect and correct versions of Fig. 8 are shown below:

Incorrect


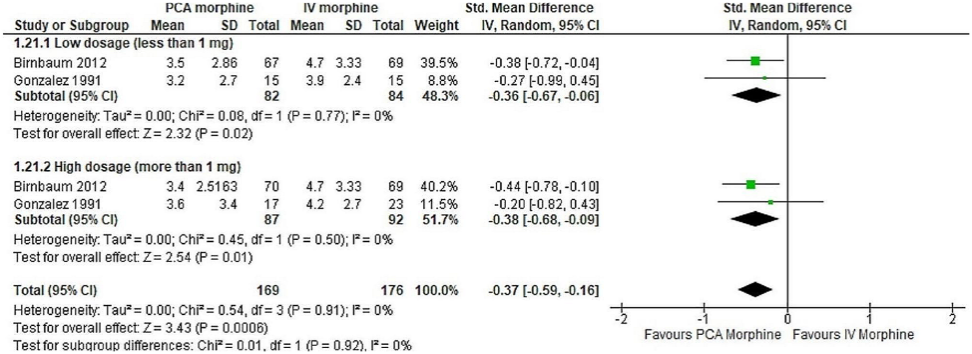


Correct

**Fig. 8:**
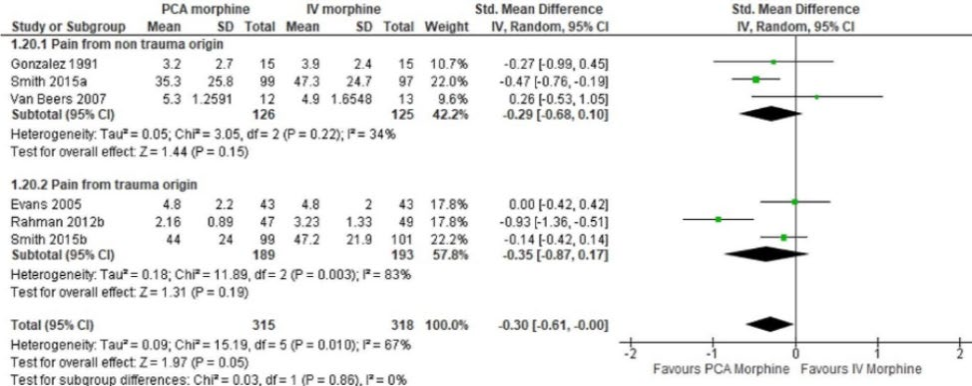
PCA morphine vs. IV morphine for the outcome of pain by origin

The original article [1] has been corrected.

## References

[1] Oon MB, Ab N, Rahman NH, Noor M (2024). Patient-controlled analgesia morphine for the management of acute pain in the emergency department: a systematic review and meta-analysis. Int J Emerg Med.

